# Cost of TB prevention and treatment in the Philippines in 2017

**DOI:** 10.5588/ijtld.21.0622

**Published:** 2022-05-01

**Authors:** T. P. J. Capeding, J. D. Rosa, H. Lam, D. G. Gaviola, A. M. C. Garfin, C. Hontiveros, L. Cunnama, Y. V. Laurence, N. Kitson, A. Vassall, S. Sweeney, I. Garcia-Baena

**Affiliations:** 1 Institute of Health Policy and Development Studies, National Institutes of Health, University of the Philippines Manila, Manila, Philippines; 2 Department of Health, National TB Control Programme, Manila, Philippines; 3 Health Economics Unit & Health Economics Division, University of Cape Town, Cape Town, South Africa; 4 Department of Global Health and Development, Centre for Health Economics in London, London School of Hygiene & Tropical Medicine, London, UK; 5 Global TB Programme, World Health Organization, Geneva, Switzerland

**Keywords:** tuberculosis (TB), cost, provider cost, Philippines, treatment support visits

## Abstract

**BACKGROUND:**

The Philippines aims to accelerate TB reduction through the provision of universally accessible and affordable services. The objectives of this paper are to estimate the costs of TB services and interventions using a health systems’ perspective, and to explore cost differences in service delivery via primary care facilities or hospitals.

**METHODS:**

Data were collected from a multi-stage stratified random sampling of 28 facilities in accordance with Global Health Cost Consortium costing standards and analysis tools. Unit costs (in US$) estimated using top-down (TD) and bottom-up (BU) approaches, are summarised following Value TB reporting standards and by broad facility type.

**RESULTS:**

Cost of delivering 32 TB services and eight interventions varied by costing method and delivery platform. Average BU costs ranged from US$0.38 for treatment support visits, US$2.5 for BCG vaccination, US$19.48 for the Xpert® MTB/RIF test to US$3,677 for MDR-TB treatment using the long regimen. Delivering TB care in hospitals was generally more costly than in primary care facilities, except for TB prevention in children and MDR-TB treatment using the long regimen.

**CONCLUSION:**

Comprehensive costing data for TB care in the Philippines are now available to aid in the design, planning, and prioritisation of delivery models to End TB.

## Abstract (fr)

**CONTEXTE:**

Les Philippines ont pour objectif d’accélérer la réduction de la TB en dispensant des services abordables et accessibles de manière universelle. Nos objectifs étaient d’estimer les coûts des services et interventions antituberculeux du point de vue des systèmes de santé, et d’analyser les différences de coûts lorsque les hôpitaux ou les centres de soins proposent ces services.

**MÉTHODES:**

Les données ont été recueillies par échantillonnage aléatoire à plusieurs degrés de 28 établissements, conformément aux outils d’analyse et normes de calcul des coûts du Global Health Cost Consortium. Les coûts unitaires (en USD), estimés à l’aide des méthodes descendante (TD) et ascendante (BU), sont résumés selon les normes du Value TB Data Collection Tool, ainsi que par type général d’établissement.

**RÉSULTATS:**

Le coût de 32 services antituberculeux et huit interventions variait en fonction de la méthode de calcul des coûts et de la plateforme dispensant ces services. Les coûts BU moyens variaient de 0,38 USD pour les consultations de soutien au traitement, 2,5 USD pour la vaccination par le BCG, 19,48 USD pour le test Xpert® MTB/RIF à 3 677 USD pour le traitement de la MDR-TB par schéma thérapeutique long. La fourniture de soins antituberculeux en hôpitaux était généralement plus chère qu’en centres de soins primaires, à l’exception de la prévention de la TB chez l’enfant et du traitement de la MDR-TB par schéma long.

**CONCLUSION:**

Les données exhaustives sur les coûts des soins antituberculeux sont désormais disponibles aux Philippines, afin d’aider à la conception, à la planification et à la priorisation des modèles de fourniture des services pour mettre fin à la TB.

The Philippines is heading towards universal health coverage (UHC), but has a high TB incidence with 599 cases per 100,000 people (range: 336–936) in 2019.^1^ TB was the fifth leading cause of mortality in that year.^2^ There are 1.8% (range: 1.3–2.6) and 28% (range: 27–29) drug-resistant TB cases estimated among new and retreatment cases respectively. Patients with drug-resistant TB accounted for 1.8% (range: 1.3–2.6) of new cases and 28% (range: 27–29) of retreatment cases. The government aims to accelerate the reduction of TB through provision of people-centred, universally accessible and affordable quality services in the Philippines.^3^

Since ensuring that all essential health services are covered under a national health coverage scheme is a key undertaking under the UHC, an up-to-date assessment of the costs of TB services and their cost drivers will contribute to better estimations of resource requirements for TB, as well as TB package reimbursement design.^4^ Progress towards UHC in the Philippines also requires the development of well-designed province and city-wide healthcare provider networks.^5^ TB service provider networks include primary care facilities (PCFs), such as rural health units and health centres, and hospitals.^6^ Differences in costs of TB services across PCFs and hospitals have not been previously estimated, and their assessment can contribute to improving the organisation of service delivery models for TB management and prevention and inform TB budget formation for the scale up of TB services in city- and province-wide provider networks.

This study aimed to estimate the costs of delivering TB services and interventions in the Philippines from a health systems’ perspective, and to contribute comprehensive data using the latest global costing standards^6^ to help inform resource allocation and planning for the effective implementation of universal healthcare.

## METHODS

Methods and tools for protocol development, cost data collection, analysis and reporting were adapted from “Costing Guidelines for Tuberculosis Interventions”,^7^ and are detailed elsewhere.^8^ Costs of delivering 32 TB services and eight interventions in the Philippines were estimated from a health provider’s perspective. Full financial and economic costs were collected retrospectively and reflected ‘real world’ implementation of interventions. Where elements of TB services were not fully implemented in health facilities at the time of data collection, they were renamed to reflect partial implementation or removed from the analysis. No start-up costs for new interventions or costs of supporting service changes were included. Estimation of above service-level costs and any research costs were excluded.

### Sampling frame and study population

The sampling frame was created from a national list of all public health facilities—private healthcare facilities regularly reporting cases to the NTP (private engaged) and private non-engaged, as of 2017. Given logistical and study budget constraints, three out of 17 regions were purposively selected based on general availability of TB interventions and services, urbanicity and presence of private sector facilities. This included Regions XI (Davao), 4B (Southwestern Tagalog Region) and III (Central Luzon), accounting for 19 of 101 million (18.7%) of the Philippines’ population, 0.7% of drug-susceptible TB notifications and 2.8% of multidrug-/rifampicin-resistant TB notifications and including 22 out of 144 cities.

The anonymised facilities were selected from these regions using multi-stage stratified random sampling. Inclusion criterion was health facilities that provided TB treatment and diagnosis. Exclusion criterion was facilities that do not report TB service provision. Facilities were categorised by urbanicity, ownership and facility type. Twenty-eight facilities from the three regions were selected for the study (Table 1, Supplementary Table S1).

**Table 1:** Characteristics and selected indicators of activity volume in 28 sampled health facilities of the Philippines, March 2018–November 2019

Facility code (region)	Facility level	Ownership	Locality	Total number of TB patients (2017)	Total beds *n*	Total outpatient visits *n*	Outpatient visits for TB *n*	Total laboratory tests *n*
PH22 (IV-B)	Primary hospital	Public	Rural	76	75	204,129	16,759	31,075
PH6 (IV-B)	Health centre	Public	Rural	12	0	1,947	1,740	68
PH16 (IV-B)	Secondary hospital	Public	Urban	79	150	242,088	23,351	170,658
PH17 (IV-B)	Community health unit	Public	Urban	242	0	16,958	12,518	6,317
PH10 (IV-B)	Community health unit	Public	Rural	184	3	20,666	10,498	24,569
PH26 (IX)	Tertiary hospital	Private for-profit	Urban	46	100	20,929	1,013	1,625
PH11 (IX)	Health centre	Public	Urban	94	0	1,472	1,229	316
PH21 (IX)	Health centre	public	Urban	200	0	19,636	7,056	4,723
PH8 (IX)	Tertiary hospital	Public	Urban	109	600	274,293	171,190	107,675
PH12 (IX)	Primary hospital	Private for-profit	Urban	NA	34	4,814	602	28,347
PH4 (IX)	Primary hospital	Public	Urban	87	35	2,244	760	15,953
PH7 (IX)	Health post/dispensary	Public	Urban	162	0	9,000	5,597	2,090
PH9 (III)	Primary hospital	Private for-profit	Rural	NA	15	4,288	22	0
PH3 (III)	Health centre	Private for-profit	Rural	4	0	1,236	29	0
PH5 (III)	Health centre	Private for-profit	Urban	NA	0	1,200	40	17
PH15 (III)	Community health unit	Public	Urban	130	0	11,776	3,173	2416
PH19 (III)	Community health unit	Public	Rural	202	0	9,255	4,061	432
PH25 (III)	Primary hospital	Private for-profit	Urban	14	35	5,362	478	100
PH24 (III)	Community health unit	Public	Rural	130	0	42,524	2,496	3,763
PH20 (III)	Community health unit	Public	Rural	146	0	29,434	21,081	1,795
PH18 (III)	Tertiary hospital	Private for-profit	Urban	42	150	30,076	1,265	126,052
PH23 (III)	Secondary hospital	Public	Rural	16	50	83,535	4,612	3,638
PH13 (III)	Primary hospital	Private for-profit	Urban	31	60	12,136	261	34,377
PH27 (III)	Tertiary hospital	Public	Urban	108	408	233,017	24,094	249,461
PH1 (III)	Tertiary hospital	Private for-profit	Urban	18	1,65	298,88	1,590	78,080
PH28 (III)	Health centre	Private for-profit	Urban	NA	0	7,300	92	3,649
PH14 (III)	Community health unit	Public	Rural	180	0	33,381	29,494	6,229
PH2	Basic laboratory (stand alone)	Private for-profit	Urban	NA	0			91

^*^ Indicates facilities provided TB services exclusively.

NA = not applicable (no patients on treatment, identification and referral of TB cases only).

### Data collection: implementing global standards with “Value TB” costing tools

Data were collected by six trained researchers (working in pairs over a 7-day period) in 28 facilities between March 2018 to November 2019 using the latest global costing standards, methods and tools, including Value TB “Data Collection”, “Data Entry” tools and checklists^7^ and Global Health Cost Consortium’s reference case.^9^ All costs were for the calendar year 2017, except for four private, non-engaged facilities, for which 2018 data were collected in 2019, when mandatory notification entered into force. In-field collection took a week, followed by another week of review prior to reporting forward to study analysts at the University of the Philippines Manila, the Philippines.

Staff time was measured using at least one, if not all the following methods: direct observation, semi-structured interviews and staff timesheets. Interviews were conducted with each key staff member to determine resource use and time spent in the previous month, while direct observation was used to collect information resource use and time spent for sampled observations (Supplementary Tables S2 and S3). Data were collected in Philippine pesos and converted to 2017 US Dollars (US$1 = PHP50.4) using the midmarket average exchange rate from 2 January to 29 December 2017. Costs in PHP were deflated using Philippines’s GDP deflator^10^ from 2018 to 2017 in case of data on private non-engaged facilities collected for the year 2018, which were then converted to US$.

### Key assumptions

Unavailable price data (e.g., furniture, equipment) was replaced with official government list prices and inflated to 2017 prices using GDP deflators (Supplementary Table S2).^10^ When actual building cost was not available, the study used estimated costs per square meter multiplied by the current market price. Wastage rates for medical supplies and consumables were assumed to be 0–5%, while for drugs this was 1–5%.

### Cost data cleaning and descriptive analysis

Data were cleaned following Value TB project standard processes described elsewhere.^6^ For each facility, unit cost per services and intervention were generated, reviewed, and pooled to generate national estimates, and then analysed using Stata v15 (Stata Corp, College Station, TX, USA). To ensure comparability and standardisation, naming conventions were applied to describe TB services. Descriptive analysis performed in Stata v15 was exported in to MS Excel (Microsoft, Redmond, WA, USA) following Value TB minimum reporting standards.

### Analysis by two broad types of facility

To understand the impact of providing care, unit costs were analysed by two broad categories of service providers: PCFs and hospitals. Original facility categories were regrouped into “PCFs”, including “health centres”, “basic laboratory (standalone), “health post/dispensary” and “community health unit” and “hospitals” encompassing primary-, secondary- and tertiary-level (district, national, general and referral) facilities. Mean costs for TB interventions for the PCF and hospital groups were estimated.

### Ethics statement and details of informed consent

Ethics approval was granted by the National Ethics Committee (NEC) of the Philippine Council for Health Research and Development (PCHRD; Metro Manila, Philippines), London School of Hygiene & Tropical Medicine Observational/Interventions Research Ethics Committee (London, UK; ref. 14680) and Department of Health Region XI Cluster Ethics Review Committee Submission for Davao Region (Davao City, the Philippines). The WHO Western Pacific Region granted ethic review exemption (ID N. 2018.1.STB).

## RESULTS

Characteristics of 28 sampled facilities, including selected indicators of activity volume, show sampled facilities had from 4 to 432 TB-affected patients, and that visits were highest at tertiary-level facilities (up to 1.2 million visits in 2017) (Table 1), where the array of TB diagnostic and monitoring services available is largest (Supplementary Table S1).

Cost of delivering 32 TB services and eight interventions varied by costing method and delivery platform (Table 2). Xpert^®^ MTB/RIF (Cepheid, Sunnyvale, CA, USA) had the highest BU cost, at US$19.5 (95% confidence interval [CI] 18–21), while a treatment support visit was costed at US$0.38 (BU) (95% CI 0.25–0.51). Cost drivers for most commonly provided TB services, including outpatient screening, visits and common diagnostic procedures are shown in the Figure. Overhead costs were a significant contributor to commonly provided TB services, ranging from 6% to 67% of the cost of all services using BU costing, with the exception of HIV rapid testing, sputum collection and Xpert testing, for which consumables contributed to most of the costs (range: 38–84% BU). Staff cost was the main cost driver for outpatient screening visits (43%). Further detailed statistics of the full list of unit costs for the 32 TB services costed are available in the Supplementary Data (Supplementary Tables S4, S5A, S5B)

**Figure:**
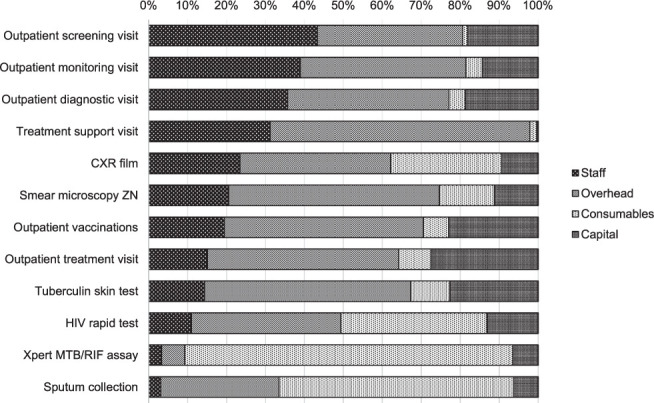
Cost drivers of bottom-up unit costs for most commonly performed TB services (%) in the Philippines (2017). CXR = chest X-ray; ZN = Ziehl-Neelsen.

**Table 2:** Unit costs of most commonly performed TB services (2017 USD) ^*^

TB services	Facilities *n*	Bottom-up		Top-down	
	
		Mean	95% CI	Mean	95% CI
Outpatient diagnostic visit	27	2.9	2.3–3.6	4.1	3.2–5.1
Outpatient screening visit	27	3.2	2.6–3.9	4.6	3.5–5.6
Outpatient treatment visit	22	2.3	1.6–3.0	3.3	2.1–4.4
Smear microscopy ZN	21	3.5	2.3–4.8	6.5	3.2–9.7
Outpatient monitoring visit	21	2.6	1.9–3.3	3.8	2.6–4.9
Outpatient vaccinations	20	2.3	1.6–2.9	3.6	2.6–4.6
HIV rapid test	15	3.4	2.9–3.9	4.6	3.8–5.4
Sputum collection	15	5.5	3.9–7.1	6.9	5.3–8.5
TST	13	4.0	2.1–5.9	7.2	2.6–12
Treatment support visit	10	0.38	0.25–0.51	0.52	0.29–0.75
CXR film	10	3.3	2.8–3.8	6.2	4.3–8.2
Xpert MTB/RIF testing	9	19.5	18.4–20.5	28.4	25.0–31.7

^*^ USD1 = PHP50.4.

USD = US dollar; CI = confidence interval; ZN = Ziehl-Neelsen; TST = tuberculin skin test; CXR = chest X-ray; PHP = Filipino peso.

Mean costs (and standard deviation [SD]) of TB intervention packages for vaccination, prevention, first and second-line treatment across all facilities are given in Table 3. Average cost of bacille Calmette-Guérin (BCG) vaccination was US$3.8 (TD) and US$2.5 (BU); cost of TB prevention in children was US$35.4 (TD) and US$24.5 (BU), and included six outpatient treatment visits and drug costs. Cost of first-line treatment, which included outpatient visits for diagnosis, medicine collection, follow-up, and an average of three sputum microscopy visits, ranged from the costlier treatment of previously treated pulmonary TB patients (PTB) at US$284 (TD) and US$250 (BU), to the treatment of new PTB adults, which cost US$146 (TD) and US$117 (BU). As expected, the standard (long) second-line treatment regimen was costlier, at US$4,000 (TD) and US$3,677 (BU) than the MDR-TB treatment (short) regimen, at US$1,382 (TD) and US$1,244 (TD). The second-line treatment package included outpatient visits, drugs, sputum microscopy and additional laboratory procedures. Further detailed statistics of the full list of unit costs for the 17 TB interventions costed are available in Supplementary Table S6.

**Table 3:** TB prevention and treatment: unit costs for intervention in sampled facilities in 2017 USD (USD1 = PHP50.4)

Intervention	Population	*n*	Bottom-up		Top-down	
	
			Mean	SD	Mean	SD
BCG vaccination	Infants	20	2.5	1.8	3.8	2.4
TB prevention	Child, isoniazid preventive therapy for 6 months	7	24.5	11.1	35.4	29.2
First-line TB treatment	Adult EPTB, new + relapse	7	137	118	151	108
	Adult PTB, new + relapse	19	117	84.6	146	96.7
	Adult PTB, previously treated	12	250	100	284	97.6
	Child PTB, new + relapse	12	138	28.5	150	33.2
Second-line TB treatment	Adult PTB, standard regimen	8	3,677	746	4,000	760
	Adult PTB, short regimen	9	1,244	327	1,382	315
	Child PTB, standard regimen	1	2,497		2,719	

USD = US dollar; PHP = Filipino peso; SD = standard deviation; BCG = bacille Calmette-Guérin; EPTB = extrapulmonary TB; PTB = pulmonary TB.

A comparison of unit costs by the two broad facility types (Table 4) indicate that BCG vaccination delivery was less costly in PCFs, at US$2.0 (BU) and US$3.3 (TD), than in hospitals, at US$3.0 (BU) and US$4.3 (TD). TB prevention in children was more costly in PCFs (US$25.2 and US$39.7 using the BU and TD approaches, respectively), than in hospitals (US$22.8 and US$24.7 using the BU and TD approaches, respectively). Delivering drug-resistant TB care using the standard treatment regimen was more costly in PCFs (BU: US$3,742; TD: US$4,089) than in hospitals (on average per episode US$3,611 [BU] or US$3,910 [TD]). Standard treatment regimen in children, observed only in one hospital, had a unit cost of US$2,497 (BU) or US$2,719 (TD) per child completing treatment. Delivering shorter regimens for MDR-TB was considerably less costly in PCFs (BU: US$1,150; TD: US$1,301) than in hospitals (BU: US$1,319; TD: US$1,447).

**Table 4:** TB prevention and treatment: unit costs for intervention delivery in primary care facilities and hospitals in 2017 USD (USD1 = PHP50.4)

Intervention	Population	Primary care facilities					Hospitals				
	
		*n*	Bottom-up		Top-down		*n*	Bottom-up		Top-down	
			
			Mean	SD	Mean	SD		Mean	SD	Mean	SD
BCG vaccination	Infants	9	2.0	0.96	3.3	2.0	11	3.0	2.2	4.3	2.7
TB prevention	Child, isoniazid preventive therapy for 6 months	5	25.2	13.5	39.7	34.6	2	22.8	0.01	24.7	0.24
First-line TB treatment	Adult EPTB, new + relapse	4	88.3	22.8	108	32.8	3	203	172	209	157
	Adult PTB, new + relapse	11	74.6	31.9	100	54.7	8	176	100	210	108
	Adult PTB, previously treated	7	200	68.7	240	80.4	5	319	102	345	91.9
	Child PTB, new + relapse	9	136	32.5	146	37.0	3	145	12.6	161	18.7
Second-line TB treatment	Adult PTB, standard regimen	4	3,742	710	4,090	666	4	3,611	884	3,910	939
	Adult PTB, short regimen	4	1,150	463	1,301	452	5	1,319	194	1,447	181
	Child PTB, standard regimen	0					1	2,497		2,719	

USD = US dollar; PHP = Filipino peso; SD = standard deviation; BCG = bacille Calmette-Guérin; EPTB = extrapulmonary TB; PTB = pulmonary TB.

## DISCUSSION

The Philippines Value TB Study was conducted to estimate mean unit costs of TB services and interventions from 28 randomly selected facilities in three purposively sampled regions using global costing standards and bottom-up and top-down methods. This is the first study in the Philippines to estimate the comprehensive cost of delivering a large range of TB services (*n* = 32) and interventions (*n* = 17). TD unit costs for both TB services and TB interventions were higher than BU cost, possibly due to efficiency gaps in service delivery; this is also supported by the finding that overhead expenses were a major driver of total costs for most TB services, which could be a potential area of focus for resource managers in terms of cost reduction.

The cost of delivering drug-resistant care in the Philippines has decreased compared to when first and last measured in 2002.^11^ The 2002 study estimated that MDR-TB treatment cost US$4,915 (adjusted to 2017 prices), higher than the TD (US$4,000) and BU (US$3,677) 2017 costs we estimated. The main cost driver for both studies were drugs, accounting for 64% in 2017 and 57% in 2002.^11^ In addition to the decreasing cost of MDR-TB drug regimens and evolution in diagnosis and care protocols, methodological differences in the two costing studies may account for the difference across time in MDR-TB treatment costs. Costs per patient for data management, contact tracing and hospitalisation were included from the 2002 estimates, which were based on a single facility, but excluded in 2017 (based on 28 facilities). We found that many of the laboratory procedures for MDR-TB patients, such as sputum culture and blood chemistry, were performed outside the facilities sampled (unlike the 2002 study, which recorded in-house laboratory testing that was reflected in the study’s unit cost per MDR-TB treatment). Drug regimens used in 2002 included p-aminosalicylic acid (PASER^®^; Jacobus Pharmaceutical Company, Princeton, NJ, USA) is no longer used in 2017.

### Intervention cost differences between the two broad facility types: hospitals and primary care facilities

Lower prevention and treatment intervention unit costs were mostly observed in PFCs than in hospitals; however, TB prevention in children using isoniazid therapy and MDR-TB treatment using the standard protocol were the two exceptions. The largest difference in intervention costs was observed in the treatment of newly diagnosed PTB, where (BU) treatment cost in hospitals was 57% higher than in PCFs. The difference in delivering standard MDR-TB treatment care through PCFs or hospitals was small (PCF 3.5% higher than in hospitals). Differences may be due to the number of average outpatient visits related to the intervention, as treatment of new PTB in hospitals required more frequent visits (and associated in-house laboratory testing), whereas the standard MDR-TB treatment delivery in PCFs required more frequent visits than in hospitals. This is consistent with findings from other studies where frequency of visits led to an increase in intervention costs.^12^ The smaller gap between PCFs and hospitals in providing MDR-TB treatment is partly explained by the higher number of visits and diagnostic procedures offered at hospitals: chest X-rays and other laboratory tests in line with TB national recommendations were more easily accessible in hospital settings.

PCFs usually outsource diagnostic and adverse events testing for TB patients, Tuberculin skin test (TST) and outpatient visits for isoniazid TB prevention therapy were more costly in the PCFs, as were staff costs, primarily because of the higher salaries of staff in PCFs compared to hospitals. Also, as the administration and support services were often provided by the same salaried staff, this drove up costs for administration and support services as well.

### What this means for the organisation of service delivery

It appears that intuitively PCFs to deliver treatment is less costly than delivering treatment services in hospitals; this is supported by findings from previous studies.^13^ We found that all but two interventions (isoniazid TB prevention therapy in children and standard MDR-TB treatment protocol) were more costly to deliver in hospitals. Our findings could be used to inform service delivery arrangements (specimen transportation or referral systems) aimed at lowering costs and improve efficiency. Options for allocating TB services where quality and efficiency are maximised could be explored, while ensuring that lower costs of care delivery do not lead to higher costs borne by TB-affected households^14^ or affect access to TB care issues for hard-to-reach populations.

### Limitations of this study and recommendations for further research

This study had some limitations. First, the sample of 28 facilities was not adequate for cost function analysis. Second, data collection was limited to TB services delivered at the facility level (and excluded above-service cost estimations). This means that outsourced nationally recommended tests, such as those used for MDR-TB treatment, were excluded for unit cost estimates as presented here. As the study deals with the health system perspective only, future research could combine our results with those from previous costing studies from a patient perspective^14^ and the results of the 2017 National TB Patient Cost Survey;^15^ this would provide a more complete picture of the variations in costs of TB services. Finally, the cost variations observed across facility type may have been due to varying quality and standards in TB service delivery. Additional research would be needed to analyse the quality and cost-effectiveness of services in PCFs and hospitals.

## CONCLUSION

For the first time, comprehensive unit cost data for TB services and major cost drivers in the Philippines are now available, allowing planners and managers of TB services to make more informed decisions. This cost evidence will assist shape future TB care delivery arrangements and identify cost-cutting options for the health system. Unit costs of TB-related services estimated in this study from a substantial sample of 28 facilities showed that prevention and treatment interventions were less costly in 2017 when delivered through PCFs.

## References

[1] World Health Organization (2020). Global tuberculosis report, 2020.

[2] World Health Organization (2021). Global health estimates: Leading causes of death. https://www.who.int/data/gho/data/themes/mortality-and-global-health-estimates/ghe-leading-causes-of-death.

[3] Official Gazette (2018). An Act Instituting Universal Health Care for All Filipinos, Prescribing Reforms in the Health Care System, and Appropriating Funds Therefor Manila, The Philippines: Official Gazette. https://www.officialgazette.gov.ph/downloads/2019/02feb/20190220-RA-11223-RRD.pdf.

[4] Laurence YV, Griffiths UK, Vassall A (2015). Costs to health services and the patient of treating tuberculosis: a systematic literature review. PharmacoEconomics.

[5] Department of Health (2019). Implementing rules and regulations of the Universal Health Care Act, 2019. https://doh.gov.ph/sites/default/files/basic-page/UHC-IRR-signed.pdf.

[6] Alva S, Cloutier S (2019). Quality of tuberculosis services assessment in the Philippines: report.

[7] Cunnama L (2019). Costing guidelines for tuberculosis interventions. https://www.who.int/tb/publications/costing_guidelines/en/.

[8] Sweeney S (2022). Costs of TB services. Approach and selected findings of a multi-country study. Int J Tuberc Lung Dis.

[9] Vassal A (2017). Reference case for estimating the costs of global health services and interventions. Global Health Cost Consortium.

[10] World Bank (2020). GDP deflator: Linked series (base year varies by country) - Philippines. https://data.worldbank.org/indicator/NY.GDP.DEFL.ZS.AD?end=2018&locations=PH&start=2017.

[11] Tupasi TE (2006). Feasibility and cost-effectiveness of treating multidrug-resistant tuberculosis: a cohort study in the Philippines. PLoS Med.

[12] van Rensburg C (2019). Cost outcome analysis of decentralized care for drug-resistant tuberculosis in Johannesburg, South Africa. PLoS One.

[13] Bada FO (2019). Cost of three models of care for drug-resistant tuberculosis patients in Nigeria. BMC Infect Dis.

[14] World Health Organization (2020). Global tuberculosis report, 2020.

[15] World Health Organization (2017). Tuberculosis patient cost surveys: a handbook.

